# Health Impacts of Estrogens in the Environment, Considering Complex Mixture Effects

**DOI:** 10.1289/ehp.10443

**Published:** 2007-08-30

**Authors:** Amy L. Filby, Teresa Neuparth, Karen L. Thorpe, Richard Owen, Tamara S. Galloway, Charles R. Tyler

**Affiliations:** 1 School of Biosciences, University of Exeter, Exeter, United Kingdom; 2 Ecotoxicology and Stress Biology Research Centre, University of Plymouth, Plymouth, United Kingdom; 3 Environment Agency, Westbury-on-Trym, Bristol, United Kingdom

**Keywords:** 17α-ethinylestradiol, environmental estrogen, fish, genotoxicity, health, immunotoxicity, metabolism, mixture effect, wastewater treatment works effluent

## Abstract

**Background:**

Environmental estrogens in wastewater treatment work (WwTW) effluents are well established as the principal cause of reproductive disruption in wild fish populations, but their possible role in the wider health effects of effluents has not been established.

**Objectives:**

We assessed the contribution of estrogens to adverse health effects induced in a model fish species by exposure to WwTW effluents and compared effects of an estrogen alone and as part of a complex mixture (i.e., spiked into effluent).

**Methods:**

Growth, genotoxic, immunotoxic, metabolic, and endocrine (feminized) responses were compared in fathead minnows (*Pimephales promelas*) exposed for 21 days to a potent estrogenic effluent, a weakly estrogenic effluent before and after spiking with a steroidal estrogen [17α-ethinyl-estradiol (EE_2_)], and to EE_2_ alone.

**Results:**

In addition to endocrine disruption, effluent exposure induced genotoxic damage, modulated immune function, and altered metabolism; many of these effects were elicited in a sex-specific manner and were proportional to the estrogenic potencies of the effluents. A key finding was that some of the responses to EE_2_ were modified when it was present in a complex mixture (i.e., spiked into effluent), suggesting that mixture effects may not be easily modeled for effluent discharges or when the chemicals impact on a diverse array of biological axes.

**Conclusion:**

These data reveal a clear link between estrogens present in effluents and diverse, adverse, and sex-related health impacts. Our findings also highlight the need for an improved understanding of interactive effects of chemical toxicants on biological systems for understanding health effects of environmental mixtures.

There is heightened concern worldwide about the impacts of endocrine-disrupting chemicals present in the aquatic environment that can alter physiological function in wildlife and humans (reviewed by Tyler et al. 1998). Effluents from wastewater treatment works (WwTWs) are a major point source of endocrine-disrupting chemicals into watercourses, and exposure has been associated with a range of reproductive impacts, particularly in fish, including the induction of intersex (Jobling et al. 1998), lowered hormone levels (Folmar et al. 1996), and reduced gamete production and fertilization capability (Jobling et al. 2002a, 2002b).

In addition to effects on reproductive development and function, exposure to WwTW effluents has been associated with wider adverse health effects, including genotoxic damage (Gravato and Santos 2003; Liney et al. 2006), immunosuppression (Hoeger et al. 2005; Liney et al. 2006; Price et al. 1997), altered activity of hepatic phase I/phase II biotransformation enzymes (Gagne et al. 2006; Gravato and Santos 2003; Hoeger et al. 2005), nephrotoxicity (Liney et al. 2006), and reduced growth/condition (Smolders et al. 2002). Moreover, some of these effects have been shown to occur at effluent concentrations considerably lower than those that cause feminization of the reproductive system (Liney et al. 2006). The potential for WwTW effluents to affect health in these ways is of significant concern. For example, immune suppression can lead to higher disease susceptibility; altered metabolic capability can lead to toxic accumulation of pollutants or production of reactive metabolites; and damage to DNA can result in embryo mortality, developmental abnormalities, and/or cancer.

There is now considerable evidence from both field and laboratory studies that the reproductive effects of WwTW effluents result from exposure to environmental estrogens present in effluents, in particular natural and synthetic steroidal estrogens [e.g., estrone, 17β-estradiol (E_2_), 17α-ethinyl-estradiol (EE_2_) (Desbrow et al. 1998)]. Studies in mammalian systems have shown that some estrogens (and/or their metabolites) also have, for example, genotoxic properties (reviewed by Roy and Liehr 1999) and immunotoxic properties (Ahmed 2000). However, the possible role(s) of steroidal estrogens as causative agents of such wider health effects associated with exposure to WwTW effluents has not been established. Furthermore, essentially nothing known health effects of steroidal contained in complex environmental mixtures, such as effluent discharges, compared with their effects when exposed as individual chemicals. This is a significant information gap given the potential for interactive biological effects of estrogens with other environmental chemical present in mixtures.

In this study, we investigated the contribution of steroidal estrogens to a suite of adverse sublethal health effects induced by exposure to WwTW effluents via two sets of experiments with a model fish species (fathead minnow; *Pimephales promelas*). The work included a comparative analysis on the biological responses induced by the steroidal estrogen EE_2_, as a single chemical and as part of a complex environmental mixture (spiked into effluent). Health effects were determined using a battery of end points indicative of growth effects [length, weight, condition factor, and hepatic expression of the gene for the somatotropic hormone insulin-like growth factor-1 (*igf1*)], genotoxicity (comet assay), immunotoxicity [differential white blood cell (WBC) count and phagocytotic activity of liver cells], and metabolic responses [hepatic ethoxy-resorufin-*O*-deethylase (EROD) activity and cytochrome P450 1a (*cyp1a*) and 3a (*cyp3a*) gene expression, as indicators of phase I bio-transformation, and glutathione *S*-transferase (*gst*) gene expression, as an indicator of phase II biotransformation]. These responses were measured alongside endocrine (feminized) responses [gonadal growth, development of secondary sex characters, the female yolk protein precursor vitellogenin (VTG), and sex steroid receptor gene expression].

## Materials and Methods

### Test species

The fathead minnows used in the exposures were bred at the University of Exeter. Fish were maintained in glass aquaria under flow-through conditions in dechlorinated water at 25 ± 1°C, with a 16-hr light:8-hr dark photoperiod. Fish were fed adult *Artemia* sp. twice daily and Ecostart 17 1.0-mm fish food pellets (Biomar Ltd., Brande, Denmark) once daily. All animals were treated humanely according to U.K. Home Office (1986) guidelines [Animals (Scientific Procedures) Act 1986].

### Test substances and dosing system

The effluents used in this study were collected from the final effluent channels at two U.K. WwTWs at weekly intervals during November/December 2005 (experiment 1) and June 2006 (experiment 2) (2,000 L/batch). The effluents were transported immediately to the laboratory, where they were transferred into a fully enclosed stainless steel holding tank cooled to 8°C. For the different batches of effluent, conductivity ranged from 1,098 to 1,166 mS/cm, pH ranged from 7.8 to 8.5, nitrate was < 5 mg/L, nitrite was < 0.2 mg/L, and ammonia ranged from 0.6 to 1.2 mg/L. EE_2_ (98% purity; lot 024K1196; Sigma, Poole, UK) was prepared as a solvent-free stock solution; during the exposures, EE_2_ was prepared every 2 days, as previously described (Filby et al. 2007). Effluent, EE_2_, and dilution water were pumped via peristalsis into glass mixing chambers and subsequently to each exposure tank (20 L volume) at a total flow rate of 30 mL/min. The exposure tanks were gently aerated at the surface to ensure that the dissolved oxygen concentrations remained > 80% of the air saturation value throughout.

### Experimental designs

We conducted two experiments. Experiment 1 was designed to investigate for effects on a range of biological end points indicative of different health effects. Fish were exposed to a WwTW final effluent previously shown to have a strong estrogenic potency [11.1–38.2 ng E_2_ equivalent/L; steroidal estrogen concentrations of 6.5–97.4 ng estrone/L, 0.7–8.8 ng E_2_/L, and < 0.5–1.5 ng EE_2_/L; nonsteroidal estrogen concentrations of 0.62–2.7 μg nonylphenol (NP)/L and 0.03–0.41 μg octylphenol/L] and that had previously been shown to induce marked feminized responses in fish (Environment Agency, in press; Liney et al. 2005, 2006; Rodgers-Gray et al. 2001). In experiment 2, we determined the roles of estrogens present in the effluent in inducing the health effects elicited in the first experiment. To determine these roles, we compared the responses of fish exposed to a WwTW final effluent previously shown to be weakly estrogenic (0.9–1.2 ng E_2_ equivalent/L; steroidal estrogen concentrations of 3.5–5.0 ng estrone/L, < 0.5–1.2 ng E_2_/L, and < 0.5–1.0 ng EE_2_/L; NP concentration of < 1.0–1.2 μg/L) (Environment Agency 2007 Environment Agency in press) with and without spiking with EE_2_ (at 10 ng/L). These responses were compared with those elicited by exposure to EE_2_ alone (at 10 ng/L).

In these experiments, duplicate tanks of sexually maturing (> 150 days posthatch) mixed-sex fathead minnows (each containing 6 males and 6 females; a total of 12 males and 12 females per treatment) were exposed to the potent estrogenic effluent (experiment 1) or the weakly estrogenic effluent without or with 10 ng/L EE_2_, or to 10 ng/L EE_2_ alone (experiment 2) under flow-through conditions for 21 days. In each experiment, duplicate tanks containing the same numbers of fish were run for dilution water as negative controls.

To confirm the estrogenic potencies of the effluents used, water samples were collected from each exposure tank regularly during the exposure periods (days 8, 14, and 21 for exposure 1; days 3, 5, 7, 14, and 21 for exposure 2), extracted onto primed C_18_ solid-phase cartridges, and analyzed for estrogenic (E_2_ equivalent) activity using the recombinant yeast estrogen screen, as described previously (Filby et al. 2007). We measured the actual concentrations of EE_2_ present in the EE_2_-dosed tanks in experiment 2 by atmospheric pressure photo ionization/liquid chromatography–mass spectrometry/time of flight (APPI-LC-MS-ToF) (Environment Agency, National Laboratory Services, Nottingham, UK).

### Fish sampling and morphometric analyses

At the end of the experiments, all fish were sacrificed by a lethal dose of anesthesia (500 mg/L MS-222 buffered to pH 7.4; Sigma) and measured for total length (millimeters), wet weight (milligrams), and condition factor (fish length as a percentage of body weight). A blood sample was collected from each fish by cardiac puncture into a chilled heparinized syringe. From each blood sample, 5 μL was removed for a differential WBC count, and a further 5 μL was removed for the comet assay (in experiment 1 only); the remainder was centrifuged at 13,000 rpm for 5 min and the plasma removed and stored at −20°C until required for quantification of plasma VTG. In the male fish, the secondary sex characters (nuptial tubercles and dorsal fat pad) were quantified, as previously described by measuring the number of nuptial tubercles, their prominence (on a scale of 1–5, spanning from the least to the most prominent), fatpad index (fatpad weight as a percentage of total body weight), and fatpad grade (the degree of development of the fatpad on a scale of 0–4, corresponding to a range spanning from poorly developed to highly developed) (Filby et al. 2007). To quantify gonadal growth, the gonads were removed from each fish and weighed for determination of gonadosomatic index (gonadal weight as a percentage of total body weight). Dissected gonads were set aside for comet assay and gene expression analyses. Livers were also dissected from each fish for the phagocytosis assay (experiment 1) and/or gene expression analyses (experiments 1 and 2). Samples for gene expression and EROD analyses were snap frozen in liquid nitrogen and stored at −80°C until use.

### VTG enzyme-linked immunosorbent assay (ELISA)

VTG was measured in the plasma of all fish using a carp VTG ELISA validated for use with fathead minnows (Tyler et al. 1999). Expression of the *vtg* gene was also measured in liver (the site of production of VTG protein) by real-time polymerase chain reaction (PCR).

### Comet assay

We used the comet assay to determine the level of DNA damage (DNA strand breaks) in gonadal cells (both experiments) and/or blood cells (experiment 1 only) of all fish. The preparation of slides for the comet assay, as well as subsequent electrophoresis and staining, were carried out as described previously by Liney et al. (2006).

### Differential WBC count and phagocytosis assay

Differential WBC counts (Stolen et al. 1990) were determined microscopically (at 100× magnification) from a smear of 5 μL blood of each fish. The counts were based on the number of each type of leukocyte (lymphocytes, granulocytes, thrombocytes, monocytes) observed in a count of 200 leukocytes. The phagocytosis assay (experiment 1 only) was performed on liver cells of all fish by measuring the uptake of zymosan particles (from *Saccharomyces cerevisae*) stained with neutral red as described by Rickwood and Galloway (2004).

### Hepatic EROD activity

To isolate S9 fractions from the liver samples, samples were rinsed in ice-cold buffer (50 mM TRIS, pH 7.4; 150 mM potassium chloride; 1 mM EDTA; 20% volume glycerol + 1 mM dithio-threitol) and homogenized (5 mL/g tissue) for 10–20 sec. Homogenates were centrifuged (9,000 × *g* for 15 min at 4°C), and the supernant S9 fraction was recovered and diluted in buffer to give a final protein concentration of 0.5–5 mg/mL. Microplate EROD analysis was carried out as described by Stagg and McIntosh (1998). We measured activity in the S9 fraction by kinetic measurement over 10 min at a controlled temperature (21°C) using a Bio-tek FL600 fluorescent plate reader (Bio-tek Instruments, Inc., Winooski, Vermont, USA) with excitation at 544 nm and emission at 584 nm. Each well contained 10 μL of S9 fraction; 320 μL of 0.1 M TRIS; 0.1 M NaCl buffer, pH 8.0; 10 μL of 2 μM 7-ethoxyresorufin; and 10 μL of 0.25 mM NADPH. An external resorufin standard curve (0–1.0 μmol/L) was used to calibrate the apparatus for conversion of fluorescence units into molar quantities using Bio-tek KC4 data analysis software (Bio-tek Instruments, Inc.); EROD activity was expressed in picomoles per minute per milligram of protein. Expression of the *cyp1a* gene was also measured in liver by real-time PCR.

### Gene expression

For the gene expression analyses, total RNA was extracted from the liver and gonadal tissue samples and reverse transcribed to cDNA as previously described (Filby and Tyler 2005). Real-time quantitative PCR was performed for fathead minnow *igf1* (GenBank AY533140; National Library of Medicine 2007), *vtg* (GenBank AF130354), *cyp1a* (GenBank AF232749), *cyp3a* (GenBank DT359255), and *gst* (GenBank AF274054) in liver, and *esr1* (estrogen receptor 1; GenBank AY775183) and *ar* (androgen receptor; GenBank AY727529) in both liver and gonad, using previously described methods (Filby and Tyler 2005). 18S ribosomal RNA (18S rRNA) was used for relative quantification because its expression did not change after any of the treatments in the tissues studied. Real-time PCR primer sequences, product sizes, annealing temperatures, standard curve slope, PCR efficiencies, and correlation coefficients are available online in Supplemental Material, Table 1 (http://www.ehponline.org/members/2007/10443/suppl.pdf).

### Data analyses

All experimental data are shown as the mean ± SE. Statistical differences (*p* < 0.05) between experimental groups were assessed by Student’s *t*-test or one-way analysis of variance (ANOVA) followed by the Holm Sidak post hoc test, or non-parametric alternatives, when necessary (SigmaStat 2.03; Jandel Scientific Software).

## Results

### Water chemistry

We confirmed the estrogenic activities of the potent and weakly estrogenic effluents by *in vitro* yeast screen as 21.3 ± 9.12 ng and 6.18 ± 0.96 ng E_2_ equivalent/L, respectively. Addition of EE_2_ to the weakly estrogenic effluent increased its estrogenic activity (as measured *in vitro*) by 1.8-fold to 11.14 ± 3.387 ng E_2_ equivalent/L. The actual concentrations of EE_2_ present in the EE_2_-dosed tanks in experiment 2 were 4.460 ± 1.000 and 11.933 ± 0.470 ng/L for the EE_2_-spiked effluent and individual EE_2_ treatments, respectively.

### Growth responses

We found no effects of the potent estrogenic effluent on any of the growth measures in experiment 1 (length, weight, condition factor, and hepatic expression of *igf1*; data not shown); therefore, these analyses were excluded from experiment 2.

### Endocrine (feminized) responses

**Figure 1:**
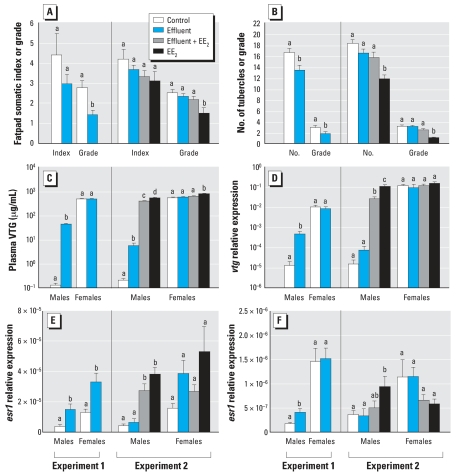
Endocrine (feminized) responses in fish exposed to a potent estrogenic WwTW effluent (experiment 1), a weakly estrogenic effluent with or without supplementation with EE_2_, or EE_2_ alone (experiment 2), quantified by (*A*) fatpad development in males, measured as fatpad index (fatpad weight as a percentage of total body weight) and fatpad grade (the degree of development of the fatpad); (*B*) tubercle development in males, measured as the number of nuptial tubercles and tubercle grade (the prominence of the tubercles); (*C*) plasma VTG concentrations; (*D*) hepatic expression of *vtg*; (*E*) hepatic expression of *esr1*; and (*F*) gonadal expression of *esr1*. Twelve males and 12 females were analyzed per treatment; data are shown as mean ± SE. Statistically significant differences between experimental groups for each sex are denoted by different letters (*p* < 0.05).

We found no effects of the effluent treatments on gonadosomatic index in either experiment (but EE_2_ alone suppressed the gonadosomatic index in males; data not shown). Exposure to the potent estrogenic effluent in experiment 1 led to a reduction in secondary sex character development in males, but this was not observed in fish exposed to the weakly estrogenic effluent in experiment 2 (Figure 1A,B). Addition of EE_2_ to the weakly estrogenic effluent in experiment 2 resulted in an apparent reduction in secondary sex character development, but this was not statistically significant, whereas EE_2_ alone induced a clear suppressive effect on these end points. In both experiments, VTG (both plasma protein and hepatic transcript) was elevated in males exposed to effluent compared with controls, although the inductions were greater for the potent estrogenic than the weakly estrogenic effluent (343-fold compared with 27-fold for plasma VTG protein, respectively; Figure 1C,D). Addition of EE_2_ to the weakly estrogenic effluent resulted in further increases in VTG in males compared with the unspiked effluent, but not to the level observed in fish exposed to EE_2_ alone. There were no effects of the effluent treatments on VTG in females (but EE_2_ alone did slightly increase VTG protein; Figure 1C). The potent estrogenic effluent, but not the weakly estrogenic effluent, up-regulated the expression of *esr1* in liver and testis (Figure 1E,F). Addition of EE_2_ to the weakly estrogenic effluent, however, did induce hepatic *esr1* in males above that of males exposed to this effluent; this effect was similar to that observed for EE_2_ alone. We found no effects of any of the effluent treatments on *ar* expression (but EE_2_ alone had a suppressive effect on *ar* in ovary) (data not shown).

### Genotoxic responses

**Figure 2:**
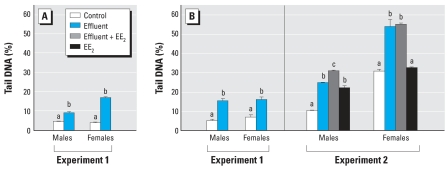
Genotoxic responses in fish exposed to a potent estrogenic WwTW effluent (experiment 1), a weakly estrogenic effluent with or without supplementation with EE_2_, or EE_2_ alone (experiment 2), quantified by the incidence of single-strand DNA breaks using the comet assay in (*A*) blood cells (experiment 1 only) and (*B*) gonadal cells (both experiments). Twelve males and 12 females were analyzed per treatment; data are shown as mean ± SE. Statistically significant differences between experimental groups for each sex are denoted by different letters (*p* < 0.05).

The incidence of single-strand DNA breaks was greater in effluent-exposed fish than in controls in both experiments (Figure 2). This occurred for both blood cells (experiment 1 only; Figure 2A) and gonadal cells (both experiments; Figure 2B). In males, addition of EE_2_ to the weakly estrogenic effluent further increased (by 1.3-fold) the genotoxic damage in gonadal cells, which was consistent with the effect of EE_2_ alone. In contrast, in females, EE_2_ alone did not cause any genotoxic damage in gonadal cells; also, addition of EE_2_ did not further increase the effect of the weakly estrogenic effluent. It should be emphasized, however, that for females in experiment 2 the background level of DNA damage in the controls was high.

### Immunotoxic responses

**Figure 3:**
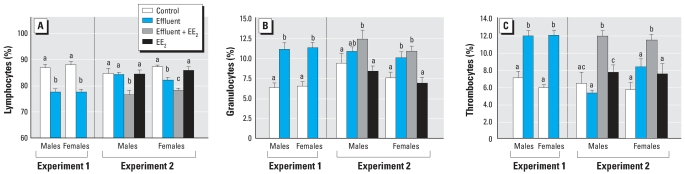
Immunotoxic responses in fish exposed to a potent estrogenic WwTW effluent (experiment 1), a weakly estrogenic effluent with or without supplementation with EE_2_, or EE_2_ alone (experiment 2), quantified by counts of lymphocytes (*A*), granulocytes (*B*), and thrombocytes (*C*) shown as percentages of total leukocytes. Twelve males and 12 females were analyzed per treatment; data are shown as mean ± SE. Statistically significant differences between experimental groups for each sex are denoted by different letters (*p* < 0.05).

Phagocytotic activity of liver cells was not affected by exposure to the potent estrogenic effluent (experiment 1; data not shown) and so was not assessed in experiment 2. In both experiments, we found a decrease in lymphocytes in effluent-exposed fish (lymphopenia; Figure 3A) and a concomitant increase in granulocytes (Figure 3B), although for the weakly estrogenic effluent these changes were only significant in females. Thrombocytes were also increased in fish exposed to the potent estrogenic effluent in experiment 1 but not to the weakly estrogenic effluent in experiment 2 (Figure 3C). In experiment 2, addition of EE_2_ to the weakly estrogenic effluent had no effect on granulocytes, but it was associated with a further reduction in lymphocyte numbers (Figure 3A) and had a stimulatory effect on thrombocytes that was not seen for this effluent alone (Figure 3C). In contrast, EE_2_ alone had no effects on WBC differentials.

### Metabolic responses

**Figure 4:**
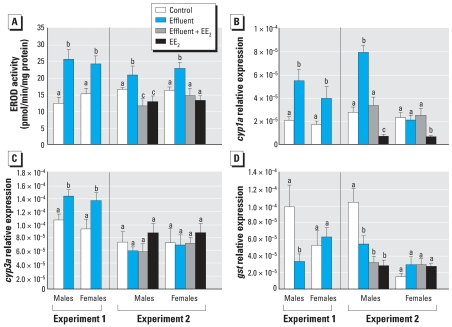
Metabolic responses in fish exposed to a potent estrogenic WwTW effluent (experiment 1), a weakly estrogenic effluent with or without supplementation with EE_2_, or EE_2_ alone (experiment 2), quantified by (*A*) hepatic EROD activity, (*B*) hepatic expression of *cyp1a*, (*C*) hepatic expression of *cyp3a*, and (*D*) hepatic expression of *gst*. Twelve males and 12 females were analyzed per treatment; data are shown as mean ± SE. Statistically significant differences between experimental groups for each sex are denoted by different letters (*p* < 0.05).

In both experiments, effluent exposure induced hepatic EROD activity (Figure 4A), and this was typically associated with parallel inductions of hepatic *cyp1a* expression (Figure 4B). In males, addition of EE_2_ to the weakly estrogenic effluent (experiment 2) resulted in lower EROD/*cyp1a* levels than in males exposed to this effluent alone, which was consistent with the inhibitory effect of EE_2_ alone on these end points. A similar effect of EE_2_ added to the weakly estrogenic effluent was observed for EROD activity in females, but EROD activity was not significantly reduced in females exposed to EE_2_ alone compared with the controls. Hepatic expression of *cyp3a* was induced only by the potent estrogenic effluent (in both males and females) and not for any of the other treatments (Figure 4C). For *gst* (Figure 4D), however, hepatic expression in males was inhibited by both effluents and to similar levels for the EE_2_-spiked effluent and EE_2_ alone in experiment 2; no effects were observed for any of the treatments in females.

## Discussion

In the present study, we have shown that steroidal estrogen present in WwTW effluents contributes to a suite of adverse (sublethal) health effects in addition to endocrine disruption, and we have further identified intriguing differences in the effects of EE_2_ on some of these responses in fish when this estrogen is administered as part of a complex mixture (effluent) versus as a single chemical. Our findings have important implications for assessing the associated health risks of estrogens as part of complex mixtures, the form in which they usually occur in the environment.

The biological effects of the effluents used in these exposures are consistent with previous research findings [including a previous study on the potent estrogenic effluent (Liney et al. 2006)] and further support the contention that exposure to WwTW effluent can lead to diverse and adverse impacts on the health of fish. The concern about health effects associated with effluent discharges in watercourses is heightened by reports in the literature that some of these effects manifest rapidly (as quickly as 4 hr after initiation of exposure) and in some cases at very low concentrations of effluent (Gravato and Santos 2003; Hoeger et al. 2005).

The greater endocrine-disrupting ability (effects on VTG, secondary sex characters, and *esr1* expression) of the WwTW effluent with the higher estrogenic potency compared with that of the more weakly estrogenic effluent, together with the enhanced endocrine-disrupting ability of the weakly estrogenic effluent after addition of EE_2_, were as expected and consistent with the fact that steroidal estrogens play a key role in disrupting sexual development in exposed fish. The less pronounced effects of EE_2_-spiked effluent compared with EE_2_ alone were in agreement with the lower than nominal concentrations of EE_2_ measured in the tanks treated with EE_2_-spiked effluent (but not EE_2_ alone), likely due to a reduced bioavailability of EE_2_ when present in effluent due to binding to particulate matter and/or degradation of EE_2_ by microorganisms present in the effluent. Of more significance in this study was that some of the wider health effects in exposed fathead minnows were also proportional to the estrogenic activity of the effluents, suggesting that this class of chemical has, at least in part, a role in the manifestation of these other observed forms of toxicity.

In male fathead minnows, addition of EE_2_ to the weakly estrogenic effluent increased its DNA-damaging effects in gonadal cells; this was consistent with the increased DNA damage observed in males exposed to EE_2_ alone compared with the controls (analysis of this nature in females was complicated by high background DNA damage). In mammalian systems, natural estrogens and xenoestrogens have been observed to cause DNA damage both *in vitro* and *in vivo* (e.g., Iso et al. 2006; Siddique et al. 2005) by way of various mechanisms, including direct effects on DNA, indirect effects via the formation of genotoxic metabolites or via induction of oxidative stress, and effects on DNA synthesis and repair, which, in some cases, are dependent on estrogen receptor activation (e.g., Iso et al. 2006). In fish, the natural estrogen E_2_ and the xenoestrogen NP have been reported to cause genotoxic effects (Teles et al. 2004, 2006), but there have been no previous studies showing these effects for EE_2_. The high induction of DNA damage observed in fathead minnows exposed to the weakly estrogenic effluent in the absence of EE_2_ supplementation nevertheless shows that other chemicals in addition to estrogens present in the effluent were likely also contributing to its genotoxicity; these chemicals may include polyaromatic hydrocarbons (PAHs), pesticides, furans, dioxins, polychlorinated biphenyls (PCBs), and/or trace metals, all of which have been shown to be present in WwTW effluents (e.g., Rule et al. 2006; Stevens et al. 2001) and are known to have genotoxic properties (e.g., Ferraro et al. 2004). Of particular concern, Butala et al. (2004) showed that when fish are exposed to estrogens and other genotoxic compounds simultaneously (as in WwTW effluents), the predominant metabolites formed from E_2_ differ from those in control fish in favor of highly reactive genotoxic catechol estrogen metabolites.

Reductions in the number of circulating lymphocytes (indicative of immunosuppression) and increases in the number of circulating granulocytes and thrombocytes (indicative of nonspecific immune activation) only occurred in fish exposed to the potent estrogenic effluent and not to the weakly estrogenic effluent. Moreover, fish exposed to the EE_2_-spiked effluent showed effects analagous to those in fish exposed to the potent estrogenic effluent although, paradoxically, EE_2_ alone had no effects on these immune parameters. As in mammals, estrogens (including EE_2_) have been demonstrated to inhibit various aspects of the immune response in fish, including phagocytosis (e.g., Watanuki et al. 2002), antibody production (e.g., Hou et al. 1999), and immune-related gene transcription (e.g., Williams et al. 2007), although reported effects have not always been consistent between studies and with different estrogens. Interestingly, the differences between the sexes in their immune responses to the weakly estrogenic effluent (a greater responsiveness in females) were further suggestive that sex steroids can strongly influence the immune system in fish, as has been established for humans (Inadera 2006). The finding of a lack of immunosuppressive effect of EE_2_ alone, but enhanced suppressive effect when EE_2_ was added to the weakly estrogenic effluent, likely reflected an interactive effect of EE_2_ on biological processes when present as a mixture with other chemicals in the effluent (Galloway and Handy 2003). For example, coexposure to EE_2_ may, for example, through an effect on absorption, distribution, metabolism, and/or signaling, facilitate the biological actions of another immunotoxic chemical present in the effluent or vice versa. Indeed, many other chemicals present in WwTW effluents, such as PCBs, PAHs, metals, and dioxins (e.g., Rule et al. 2006; Stevens et al. 2001), possess immunotoxicant properties (reviewed by Inadera 2006). This finding emphasizes our lack of knowledge of the interactive effects on biological systems of chemicals that are part of complex mixtures in environmental samples. This finding also highlights further difficulties in extrapolating from chemical effects derived from single chemical exposures to effects of the same chemical as part of a complex mixture, and especially when chemicals (such as estrogens) induce a wide range of biological effects.

Inhibition of the phase II biotransformation enzyme *gst* in males was proportional to the level of estrogenic activity in the effluents studied. GSTs detoxify a wide range of endogenous and exogenous molecules, including xenobiotics, via conjugation with the tri-peptide glutathione (Armstrong 1997). The *gst* isoform targeted in this study is particularly involved in protecting against oxidative stress (Doi et al. 2004). Hepatic GST mRNA, protein, and activity were all down-regulated by estrogens (including E_2_, EE_2_, and NP) in other studies with male or juvenile fish of unknown sex (e.g., Hughes and Gallagher 2004; Vaccaro et al. 2005). As for the genotoxic effects, however, the strong inhibitory effect of the weakly estrogenic effluent alone (without EE_2_) on *gst* levels in males suggests that other chemicals present in the effluent may have also contributed to this observed response. The lack of treatment effects on *gst* expression in females may relate to their higher levels of endogenous estrogen that, in turn, are likely to account for the lower *gst* levels observed in females compared with males, and may render them less sensitive to induction/inhibition by exogenous chemicals.

An antagonistic effect of estrogen on effluent-stimulated CYP1A activities [presumably due to the presence of aryl hydrocarbon receptor (AhR) agonists such as PAHs, PCBs, and dioxins] is in agreement with previous evidence for inhibitory effects of estrogens on both basal and AhR-induced CYP1A activities in fish tissues (e.g., Mdegela et al. 2006; Navas and Segner 2000). In fish, this effect is mediated through an estrogen receptor–dependent mechanism (Navas and Segner 2001), as is the case in mammals where estrogens decrease *cyp1a* expression by altering recruitment of the transcription factor nuclear factor-1 to the AhR, a process that normally increases *cyp1a* transcription (Ricci et al. 1999). Importantly, the observed antagonism of effluent-induced CYP1A by the added EE_2_ may result in a compromised detoxification capacity of fish exposed to potent estrogenic effluents. Moreover, as recently highlighted by Kirby et al. (2007), the simultaneous presence of CYP1A inhibitors (estrogens) and inducers (PAHs, PCBs, and dioxins) within effluents has important implications for the use of CYP1A-related end points as biomarkers of PAH exposure in environmental monitoring programs, because data may underestimate effects if estrogen-mediated CYP1A suppression is occurring in wild populations. Interestingly, as for *gst*, these findings imply that higher endogenous estrogen levels in females may be responsible for the less pronounced treatment effect observed on EROD activity in this sex.

The lack of any treatment effects on hepatic *cyp3a* in experiment 2 implies that the effluent-mediated induction of this gene in experiment 1 was likely due to chemical(s) other than estrogens that were specific to (or in sufficient concentrations to induce an effect in) the potent estrogenic effluent. In contrast with our data, there is some evidence in the literature for regulation of fish CYP3As by estrogens, however, generally in an inhibitory, rather than a stimulatory, manner (e.g., Arukwe et al. 1997; Buhler et al. 2000). Indeed, in humans EE_2_ is known to be a mechanism-based inactivator of the key CYP3A isoform CYP3A4, which may function to decrease EE_2_ metabolism because CYP3A4 is the main enzyme involved in EE_2_ oxidation (Lin et al. 2002). Interestingly, alkylphenols such as NP (which are also xenoestrogens) induce CYP3A and CYP3A-mediated testosterone 6β-hydroxylation in some fish (e.g., Baldwin et al. 2005; Meucci and Arukwe 2006), likely via activation of the pregnane X receptor, which mediates the effects of many xenobiotics and steroids on this enzyme in mammals (Meucci and Arukwe 2006). Moreover, a number of pharmaceuticals and personal care products identified in a municipal effluent were observed to induce CYP3A-related activity in trout hepatocytes (Gagne et al. 2006); thus, one possible explanation for CYP3A induction by the potent estrogenic effluent may have been higher concentrations of alkylphenols or pharmaceuticals and personal care products in this effluent.

## Conclusions

These data further highlight the diverse health implications of WwTW effluents for exposed organisms, highlight the sex-specific nature of some of these effects, and reveal for the first time a clear association between estrogens present in effluents and some of the observed adverse effects. We have demonstrated that the estrogen EE_2_, when part of a complex mixture, can impact differently on measures of health compared with when it is exposed as a single chemical. Thus, the effects of estrogens administered alone cannot always predict their effects in complex environmental mixtures, and these interactive effects have far-reaching implications for the use of some biomarkers (such as EROD) in environmental monitoring. A greater understanding of the mechanisms of interactive chemical effects is essential to fully understand the impacts of environmental mixtures like effluents for exposed organisms.
